# Independent origins of resistance or susceptibility of parasitic wasps to a defensive symbiont

**DOI:** 10.1002/ece3.2085

**Published:** 2016-03-16

**Authors:** Mariana Mateos, Lauryn Winter, Caitlyn Winter, Victor M. Higareda‐Alvear, Esperanza Martinez‐Romero, Jialei Xie

**Affiliations:** ^1^ Department of Wildlife and Fisheries SciencesTexas A&M UniversityCollege StationTexas; ^2^ Centro de Ciencias GenomicasUniversidad Nacional Autonoma de MexicoCuernavacaMorelosMexico

**Keywords:** Braconidae, defensive mutualism, *Drosophila melanogaster*, Figitidae, heritable endosymbiont, Mollicutes

## Abstract

Insect microbe associations are diverse, widespread, and influential. Among the fitness effects of microbes on their hosts, defense against natural enemies is increasingly recognized as ubiquitous, particularly among those associations involving heritable, yet facultative, bacteria. Protective mutualisms generate complex ecological and coevolutionary dynamics that are only beginning to be elucidated. These depend in part on the degree to which symbiont‐mediated protection exhibits specificity to one or more members of the natural enemy community. Recent findings in a well‐studied defensive mutualism system (i.e., aphids, bacteria, parasitoid wasps) reveal repeated instances of evolution of susceptibility or resistance to defensive bacteria by parasitoids. This study searched for similar patterns in an emerging model system for defensive mutualisms: the interaction of *Drosophila*, bacteria in the genus *Spiroplasma*, and wasps that parasitize larval stages of *Drosophila*. Previous work indicated that three divergent species of parasitic wasps are strongly inhibited by the presence of *Spiroplasma* in three divergent species of *Drosophila*, including *D. melanogaster*. The results of this study uncovered two additional wasp species that are susceptible to *Spiroplasma* and two that are unaffected by *Spiroplasma*, implying at least two instances of loss or gain of susceptibility to *Spiroplasma* among larval parasitoids of *Drosophila*.

## Introduction

Research conducted over the last two decades, aided by the availability of molecular tools, has revealed that insects engage in diverse, intimate and influential interactions with microbes (Douglas 2015). Among these, maternally inherited bacteria (often noncultivable) are common in many insect lineages, either as obligate beneficial partners (typically nutritional mutualists) or as facultative symbionts that persist by manipulating host reproduction to their own benefit and/or by conferring a fitness advantage to their hosts. Such fitness benefits can be context‐dependent and come in the form of resistance to abiotic stresses (e.g., heat tolerance) or protection against an array of natural enemies (e.g., viruses, fungi, parasitic nematodes and parasitoid wasps; Hamilton and Perlman 2013; Oliver et al. 2014). Such defensive associations can set the stage for complex ecological (Kwiatkowski and Vorburger 2012) and coevolutionary (Kwiatkowski et al. 2012) dynamics involving hosts, defensive symbionts, and natural enemies. Understanding these dynamics requires, among others, knowledge on the extent of natural enemies against which a defensive symbiont is able to protect (Vorburger 2014).

One of the best‐studied defensive symbiosis systems involves aphids (several species including the pea aphid *Acyrthosiphon pisum*), the gammaproteobacterium *Hamiltonella defensa* (several strains; although other bacterial lineages are also reported to confer defense; reviewed in Vorburger 2014), and parasitic wasps belonging to two families (Braconidae and Aphelinidae). Studies on aphid defensive symbioses have uncovered features of coevolutionary dynamics at the microevolutionary level, including evidence for effective selection on parasitoid counter‐adaptation (Dion et al. 2011; Rouchet and Vorburger 2014), and plastic behavioral responses by parasitoids to protective symbionts (Oliver et al. 2012; Lukasik et al. 2013). Recent research has also reported the existence of aphid parasitoid species that are not susceptible to otherwise protective symbionts (Asplen et al. 2014; Cayetano and Vorburger 2015; McLean and Godfray 2015), implying the repeated evolution of resistance (or susceptibility) to defensive microbes by parasitoids. Whether this phenomenon occurs in the other emerging model system for defensive symbiosis against parasitoids, the *Drosophila–Spiroplasma* association, has not been addressed.

Members of the genus *Spiroplasma* (class Mollicutes) include several maternally inherited bacteria of *Drosophila* and other insects, as well as many horizontally transmitted symbionts (including numerous pathogens) of diverse arthropods and plants (reviewed in Bolaños et al. 2015). Nineteen species of *Drosophila* are reported to harbor *Spiroplasma*, but infection prevalence varies by species and population (Watts et al. 2009; Haselkorn 2010; Jaenike et al. 2010a; unpublished data). Several *Spiroplasma* lineages associated with *Drosophila* are reproductive parasites (killing the sons of infected females) that occur at relatively low frequencies ~1–17% (Montenegro et al. 2005; Ventura et al. 2012). Nonmale killing *Spiroplasma*, however, can achieve intermediate to very high prevalence >50–85% (Haselkorn 2010; Jaenike et al. 2010b). Numerous studies on the *Drosophila*–*Spiroplasma* association have evaluated the occurrence and strength of the different forces that influence infection frequencies, which include the rates/modes of vertical (and horizontal) transmission, the benefit to the symbiont derived from the reproductive manipulation, and fitness benefits/costs of harboring the symbiont (e.g., Kageyama et al. 2006; Jaenike et al. 2007, 2010b; Martins et al. 2010; Herren et al. 2013; Xie et al. 2015).

The *Drosophila*–*Spiroplasma* system has garnered recent attention in the context of defense against natural enemies. The naturally occurring *Spiroplasma* strains associated with three distantly related species of *Drosophila* (*D. neotestacea* and *D. hydei* from the subgenus *Drosophila* and *D. melanogaster* from the subgenus *Sophophora*) are detrimental to natural enemies of their hosts. In *D. hydei*,* Spiroplasma* strain “*Shy1*” increases larva‐to‐adult survival of flies attacked by the larval parasitoid wasp *Leptopilina heterotoma* (Xie et al. 2010). In *D. melanogaster*,* Spiroplasma* strain MSRO (which is a male killer) also improves larva‐to‐adult survival of flies attacked by *L. heterotoma*,* L. boulardi*, and *Asobara tabida* (Paredes‐Escobar 2014; Xie et al. 2014). In the mycophagous fly *D. neotestacea*,* Spiroplasma* strain “*Sneo”* restores fertility in females parasitized by the sterilizing nematode *Howardula aoronymnphium* (Jaenike et al. 2010b) and also enhances larva‐to‐adult survival of flies attacked by *L. heterotoma* (Haselkorn and Jaenike 2015). All three of the *Spiroplasma* strains known to protect against parasitic wasps (and the nematode) belong to the poulsonii lineage, which is one of four *Drosophila*‐associated clades that represent independent invasions of *Drosophila* (Haselkorn et al. 2009).

The degree to which *Spiroplasma* rescues flies that have been attacked by wasps varies widely by host species and possibly by wasp strain, *Spiroplasma* strain, and experimental conditions (see Discussion). Nonetheless, the presence of *Spiroplasma* effectively blocks wasp development by a hitherto unknown mechanism that involves suppression of wasp larval growth in the two parasitoids examined to date: *L. heterotoma* and *L. boulardi* (Xie et al. 2011, 2014; Paredes et al. 2015). These observations suggest that members of the poulsonii clade are capable of preventing successful development of three divergent wasp species, representing the two families that parasitize larvae of *Drosophila* (Braconidae and Figitidae). Nevertheless, many more species of larval parasitoids attack members of *Drosophila* (Carton et al. 1986; Wachi et al. 2015), raising the question as to whether susceptibility to *Spiroplasma* (poulsonii clade, at least) is a universal feature of larval parasitoids of *Drosophila*. Knowledge on the degree of generality or specificity of the *Spiroplasma* wasp interference mechanism will offer insight into the selective pressures acting on all members of this interaction and perhaps contribute to a more comprehensive view of the forces that drive *Spiroplasma* prevalence in natural populations. This study examined the effect of *Spiroplasma* strain MSRO on larva‐to‐adult survival of *D. melanogaster* and on wasp success, when flies are subjected to oviposition by one of five wasp species not examined to date: one braconid (*Asobara japonica*); and four figitids (*L. victoriae*,* L. guineaensis*,* Ganaspis xanthopoda*, and *G. *sp.).

## Materials and Methods

### Insect sources and endosymbiont treatments

**Figure 1:**
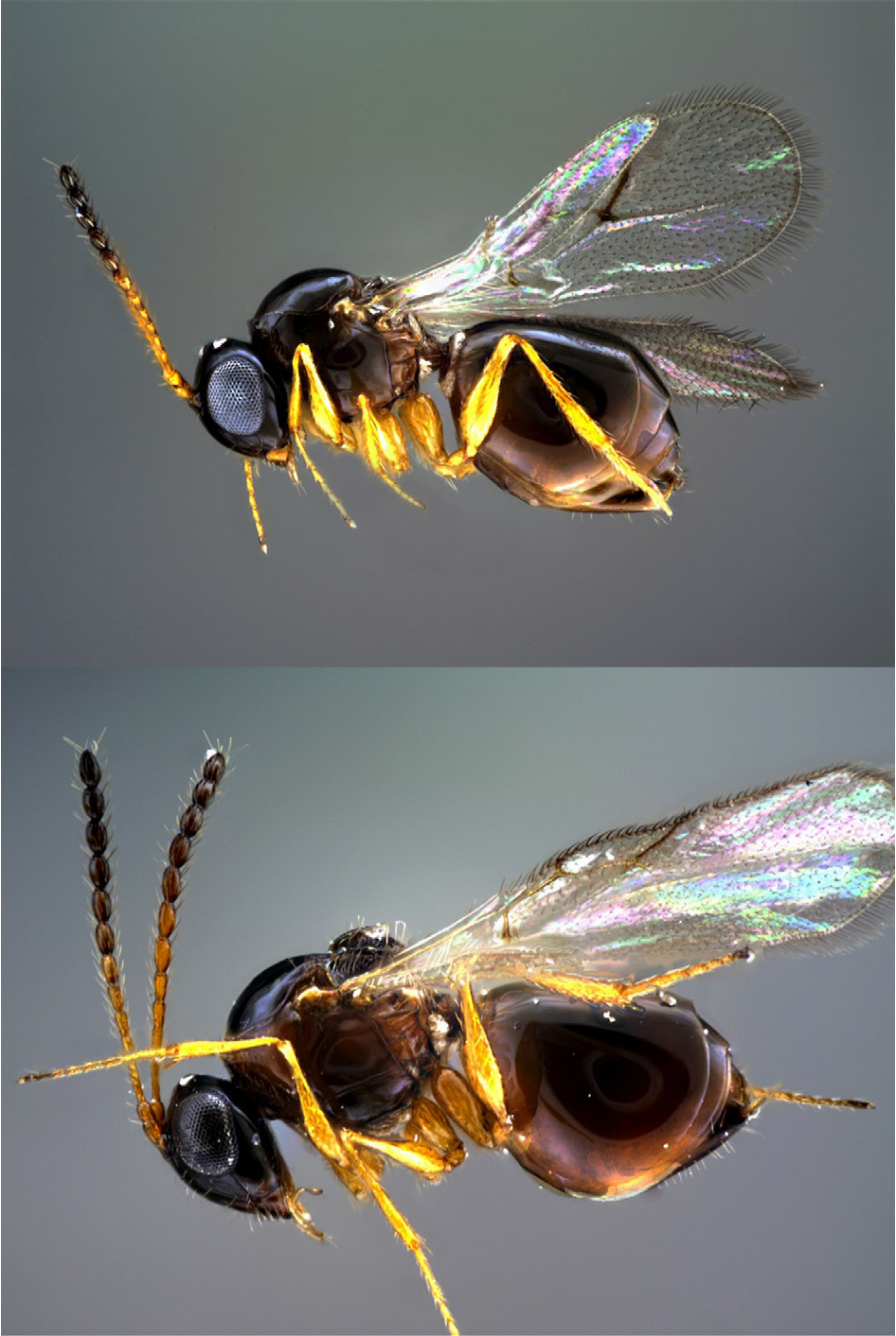
Females of two figitid wasps examined. Top *Leptopilina victoriae* (strain LvHaw). Bottom: *Leptopilina guineaensis* (strain LgG500 or LgCAM). Photographs by Matthew Buffington.

We used three *Spiroplasma*‐infected and *Spiroplasma*‐free isofemale lines of *D. melanogaster* previously established by Xie et al. (2014), via hemolymph transfer from *D. melanogaster* strain Red 42, which harbors *Spiroplasma* strain MSRO, originally collected in Campinas, Brazil (Montenegro et al. 2000). Although the original isofemale lines harbored *Wolbachia*, another heritable bacterium, *Wolbachia* was removed via antibiotic treatment several generations prior to the experimental procedures (see Xie et al. 2014). The *Spiroplasma*‐infected and free treatments were subjected to the following parasitoid wasp treatments: the figitids *L. guineaensis* (strain LgG500), *L. victoriae* (strain LvHaw), *G. xanthopoda* (strain GxHaw), and *G. *sp. (G1F1; all female); and the braconid *A. japonica* (AjJap; all female). Female specimens of the figitid species used are shown in Figure 1. The wasp strains correspond to those in Kacsoh and Schlenke (2012).

### Fitness assays

We performed five replicates for each combined treatment: two *Spiroplasma* infection states × six wasp treatments × three isolines (= 180 total replicates). Each replicate consisted of a mating/oviposition group (three females plus six males). Females were <15 days old; males were from the same isoline. Mating groups were allowed to mate and oviposit on standard cornmeal vials for two days, after which they were transferred to a fresh food vial. Approximately, 40 first/second instar larvae (~2 days old) per vial were collected and transferred into a fresh vial. Each vial was subjected to one of the following six wasp treatments: no‐wasp control; LgG500; LvHaw; GxHaw; G1F1; or AjJap. Five ~3‐day‐old wasps (which were allowed to oviposit on *D. melanogaster* prior, and thus, were “experienced”) were added per vial and allowed to oviposit for 2 days. Upon removal of wasps, ten larvae were removed from each vial and dissected to examine wasp oviposition (i.e., presence/absence of one or more wasp eggs or larvae). To ensure equivalent conditions, 10 larvae were also removed (and discarded) from the no‐wasp control vials. Only vials with 70% or more of larvae parasitized by wasps were retained (proportion of larvae parasitized per replicate is available in DataDryad submission). For each vial, we recorded the number of starting fly larvae, puparia, emerging flies, and emerging wasps. *Spiroplasma* infection status of the three mothers used in each replicate was examined by the *Spiroplasma*‐specific PCR assays described in Xie et al. (2010). Only replicates for which all three mothers had the expected *Spiroplasma* infection status were used in the analyses.

### Statistical analyses

We used JMP 11.2.0 (SAS Institute Inc., Cary, NC) to generate the results graphs. We used SAS Enterprise Guide version 7.1 statistical package (SAS Institute Inc.) to fit a generalized linear mixed model with a binomial distribution of the raw data for: (1) number of emerging adult flies/initial number of fly larvae (i.e., larva‐to‐adult fly survival rate); (2) number of pupae/initial number of fly larvae (i.e., larva‐to‐pupa fly survival rate); (3) number of emerging adult wasps/initial number of fly larvae (i.e., “larva‐to‐adult wasp survival rate”); and (4) number of failed pupae/total pupae (pupal failure). The independent variables were *Spiroplasma* infection status (fixed) and fly strain (isoline, random). These analyses were performed for each wasp treatment separately.

The specific SAS models/assumptions for each dependent variable are shown in Table S1. In general, if one category contained none to few observations (e.g., zero larva‐to‐adult fly survival), we implemented a logistic regression with a penalized likelihood (Firth method) (King and Zeng 2001). Otherwise, we attempted the generalized linear mixed model, including a COVTEST for the random factor isoline. If the output indicated that these analyses did not converge or that the G‐matrix was not positive definite, we implemented an analysis disregarding the isoline factor (for details and SAS commands, see Data S1 and Table S1).

## Results

**Figure 2:**
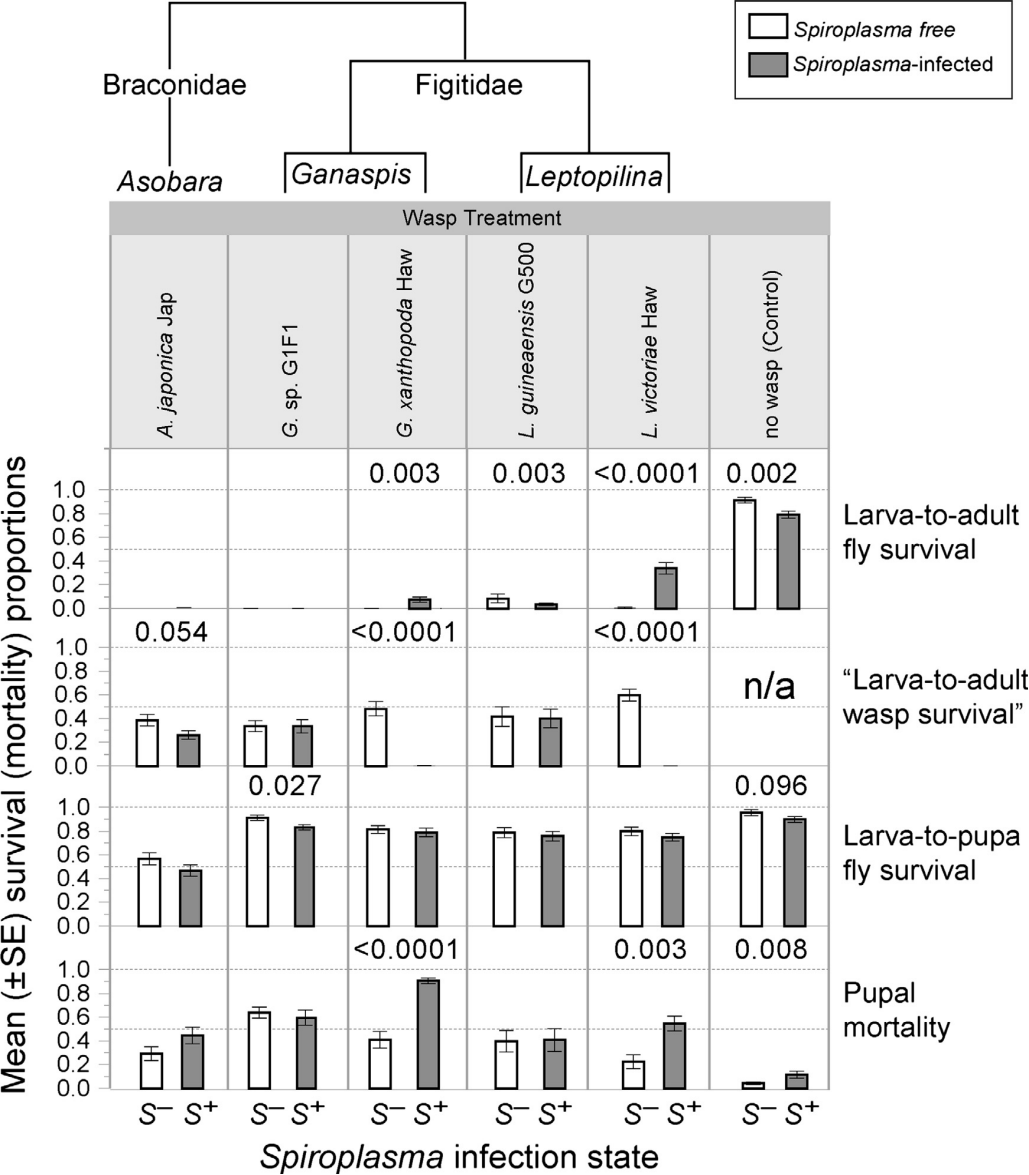
Fitness effects of *Spiroplasma* MSRO in the presence and absence of five wasp species representing three genera from two families. Mean ± Standard Error for four survival/mortality measures. Open bars = *Spiroplasma*‐free (*S* ^−^) treatments; Gray bars = *Spiroplasma*‐infected (*S* ^+^) treatments. The cladogram above indicates phylogenetic relationships (based on Kacsoh and Schlenke 2012). *P*‐values for significant (*P* < 0.05) or borderline nonsignificant effects of *Spiroplasma* infection are shown. Dashed horizontal lines = 50 and 100% *Y*‐axis values.

The data generated in this study have been deposited in Dryad under accession number doi: 10.5061/dryad.fb40c. Wasp oviposition (measured as number of fly larvae containing one or more wasp egg/larva, in a subsample of larvae from each replicate vial) was close to 100% in all replicates (range 70–100%; mean per treatment >94%). In the absence of parasitoid wasps, mean fly larva‐to‐adult survival was significantly greater in the absence of *Spiroplasma* (mean = 84%) than in the presence of *Spiroplasma* (mean = 73%) (Fig. 2), implying a slightly detrimental effect of *Spiroplasma*. Fly larva‐to‐pupa and pupa‐to‐adult (inferred from pupal failure) survival were both negatively affected by *Spiroplasma* in the absence of wasps (significant and borderline nonsignificant, respectively; Fig. 2 and Table S1).

*Spiroplasma* had no effect on the fly larva‐to‐adult survival in the presence of the braconid wasp *A. japonica*, whose parasitism caused essentially 100% fly mortality. There was, however, a slightly negative effect of *Spiroplasma* on the success of *A. japonica* (nonsignificant; *P *< 0.054), measured as the number of emerging adult wasps over the number of initial fly larvae.

The effect of *Spiroplasma* on the host–parasitoid outcome in the presence of figitid wasps was quite variable. Success of *G. *sp. G1F1 and *L. guineaensis* LgG500 was unaffected by *Spiroplasma* infection (~33% and 40% of fly larvae produced a wasp; respectively). Accordingly, survival of flies was not enhanced and appeared to be negatively affected by *Spiroplasma* in the presence of *L. guineaensis*. In contrast, *Spiroplasma* infection was highly detrimental to both *G*. *xanthopoda* and *L. victoriae*. The success of *G. xanthopoda* and *L. victoriae* in the absence of *Spiroplasma* was 48% and 60%, respectively, compared to <1% in the presence of *Spiroplasma*. The proportion of flies surviving the attack of *G. xanthopoda* or *L. victoriae* was significantly greater in the presence of *Spiroplasma*, but the *Spiroplasma*‐mediated rescue of flies was much higher when flies were attacked by *L. victoriae* (increased from <1% to ~34%) than when flies were attacked by *G. xanthopoda* (increased from 0% to ~8%), in which most of the mortality occurred at the pupal stage (i.e., neither fly nor wasp survived).

## Discussion

**Figure 3:**
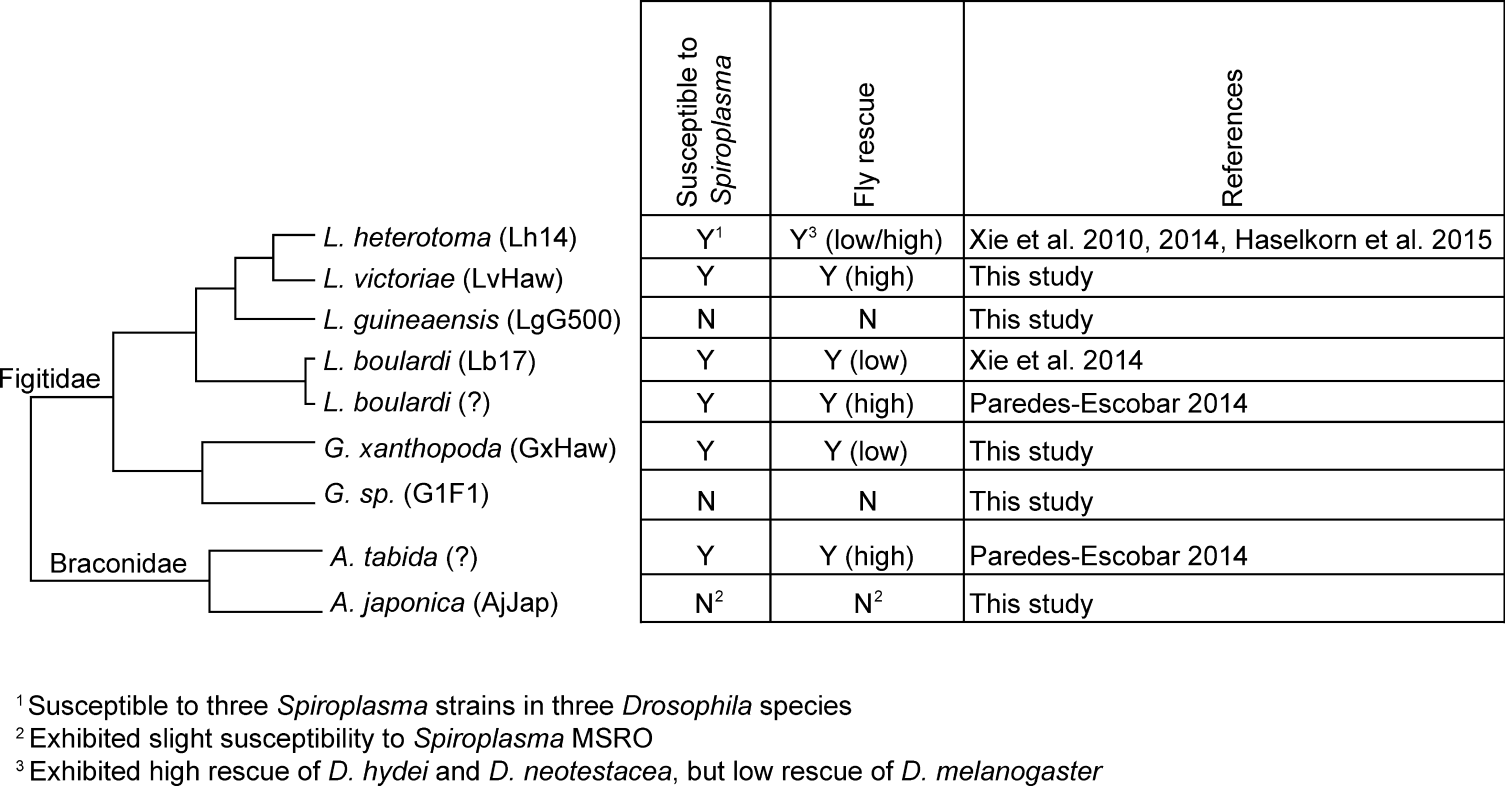
Susceptibility/resistance to *Spiroplasma* by eight species of parasitoids that attack *Drosophila*. The degree of fly rescue by *Spiroplasma* is also indicated. The phylogenetic relationships of the parasitoids are based on Kacsoh and Schlenke (2012).

Previous studies that examined the effect of *Spiroplasma* (poulsonii clade) on *Drosophila*‐parasitoid outcomes revealed that *Spiroplasma* prevents the successful development of two species of Figitidae (*L. boulardi* and *L. heterotoma*) and one species of Braconidae (*A. tabida*) in *D. melanogaster*,* D. neotestacea*, and *D. hydei* (Xie et al. 2010, 2014; Paredes‐Escobar 2014; Haselkorn and Jaenike 2015). The ability of *Spiroplasma* (poulsonii clade) to strongly inhibit members of the two families of larval parasitoids (and a sterilizing nematode) that utilize *Drosophila* as hosts was suggestive that this clade of *Spiroplasma* might be able to generally suppress *Drosophila* larval parasitoids. The present study reveals that susceptibility of *Drosophila* larval parasitoids to *Spiroplasma* is not universal. Figure 3 summarizes the current state of knowledge on the susceptibility of wasps to *Spiroplasma* (poulsonii clade) in *Drosophila*. Of the eight larval parasitoid species examined to date, five are clearly susceptible to *Spiroplasma*, two are not, and for one, *A. japonica*, our results are inconclusive. With the current patterns and sampling, it is not possible to infer the ancestral “susceptibility to *Spiroplasma*” state for the figitid and braconid parasitoids of *Drosophila,* but the patterns reveal that susceptibility or resistance to *Spiroplasma* has likely evolved at least twice in Figitidae – once in *Leptopilina* and once in *Ganaspis* – and possibly once in Braconidae, if *A. japonica* is assumed to be resistant.

A similar pattern of closely related taxa exhibiting differences in susceptibility to a defensive symbiont has recently been reported in aphid parasitoids, which belong to one of two families: Braconidae (represented by the subfamily Aphidiinae) and Aphelinidae. Although members of the three braconid genera that parasitize aphids examined to date exhibit susceptibility to at least one strain of *H. defensa*, two genera contain each at least one species that is unaffected by *H. defensa* (Asplen et al. 2014). Likewise, within Aphelinidae, of the two species examined to date (genus *Aphelinus*), one is resistant and one is susceptible to *H. defensa* (Cayetano and Vorburger 2015; McLean and Godfray 2015). Similarly, the susceptibility of *Wolbachia w*Mel‐infected mosquitos to Dengue virus varies according to virus serotype (Ferguson et al. 2015). Together, these findings suggest that other defensive mutualistic associations will likely involve variation in susceptibility among closely related natural enemies. A caveat of the present study is that only one strain per parasitoid species was examined. Future work might uncover intraspecific variation in susceptibility of parasitoids to *Spiroplasma*. Evidence for intraspecific variation in susceptibility to protective symbionts has been reported for two species of aphid parasitoids. Rouchet and Vorburger (2012) detected variation among strains of the wasp *Lysiphlebus fabarum* parasitizing the black bean aphid infected with *H. defensa*. In addition, Rouchet and Vorburger (2014) and Dion et al. (2011), respectively, successfully selected for reduced susceptibility to *H. defensa* in the parasitoids *L. fabarum* and *Aphidius ervi*.

Not all host–symbiont–parasitoid combinations where the parasitoid is killed by the presence of *Spiroplasma*, lead to substantial fly rescue; that is, the outcome that would directly benefit the prevalence of *Spiroplasma*. In such cases, the most common outcome is death of both fly and wasp at the pupal stage. Previous studies indicated five combinations that resulted in high fly rescue: *D. hydei‐Shy1‐L. heterotoma* (Xie et al. 2010), *D. neotestacea‐Sneo‐L. heterotoma* (Haselkorn and Jaenike 2015), *D. melanogaster‐*MSRO*‐A. tabida* (Paredes‐Escobar 2014), and the *D. melanogaster‐*MSRO*‐L. boulardi* combination of Paredes‐Escobar (2014; observed in both Canton S and Oregon R strains of *D. melanogaster*). Our results of *D. melanogaster*‐MSRO‐*L. victoriae* bring the total number of combinations entailing high fly rescue to six. In contrast, two combinations were previously reported to lead to low fly rescue: *D. melanogaster‐*MSRO*‐L. heterotoma* and the *D. melanogaster‐*MSRO*‐L. boulardi* combination of Xie et al. (2014; in *D. melanogaster* isofemale lines established from southern Mexico). Our present findings on *G. xanthopoda* raise to three the number of combinations involving low fly rescue despite effective wasp killing.

The reasons why *Spiroplasma* fails to rescue the host in a substantial manner for particular host–symbiont–parasitoid combinations are unknown, but could be related to the timing of wasp death (e.g., if the wasp is killed relatively late, the damage caused to the host might be irreparable). In line with this, the adult longevity and fecundity of *Spiroplasma‐*rescued *D. hydei* that were parasitized by *L. heterotoma* is lower than that of their counterparts unexposed to wasps (Xie et al. 2011), but *Spiroplasma*‐mediated protection remains advantageous, as indicated by a rapid increase in *Spiroplasma* prevalence, at least under laboratory conditions of high wasp parasitism (Xie et al. 2015). What is most intriguing is the discrepancy between two previous studies in the degree of fly rescue observed for the *D. melanogaster‐*MSRO*‐L. boulardi* combination. Whereas in Paredes‐Escobar (2014), fly survival of *L. boulardi‐*attacked flies increased from <5% to ~60% due to *Spiroplasma*, Xie et al. (2014) reported a very modest corresponding increase of <1% to 3.28%. This was observed despite similar levels of wasp success (i.e., ~70%) in the absence of *Spiroplasma* for both studies. Nonetheless, although the “virulence” of the different *L. boulardi* backgrounds used in the two studies may be similar, their interactions with host/symbiont/environment might differ. The different fly rescue of the two studies is unlikely attributable to the *D. melanogaster* genetic background alone, because Paredes‐Escobar (2014) obtained high fly rescue with the Canton S background, whereas we obtain low fly rescue with the same background (unpublished data). Experimental conditions of both studies appear to be similar (i.e., both conducted at 25°C, as well as similar fly and wasp densities and exposure times). The differences between the two studies could be due to the *Spiroplasma* strains used, as they differ in their geographical origins (Uganda vs. Brazil), and at one of the genes compared to date (9 of 800 bp at the p58 locus; Pool et al. 2006). Our present findings of high fly rescue by the Brazil‐*Spiroplasma* against *L. victoriae*, however, indicate that this combination of fly strain‐*Spiroplasma* strain‐experimental conditions can lead to substantial fly rescue. Further research exploring the interactions of host background, *Spiroplasma* background, wasp background, wasp symbionts/viruses (e.g., Fytrou et al. 2006; Furihata et al. 2015), and environmental conditions (e.g., temperature; Bensadia et al. 2006) is needed for a comprehensive understanding of the factors that lead to differential fly rescue, and ultimately influence symbiont prevalence.

Variation in susceptibility to defensive symbionts by different species of parasitoids of the same or closely related hosts has implications for ecological and evolutionary dynamics. The benefit that a symbiont provides will therefore depend on the local community of natural enemies. The dynamics will also be influenced by costs associated with symbiont infection. In *Drosophila*, evidence of relatively weak costs associated with *Spiroplasma* infection has been reported. In contrast to aphids infected with *H. defensa* (Oliver et al. 2008), the prevalence of *Spiroplasma* does not diminish in laboratory populations of *D. neotestacea* and *D. hydei* lacking the natural enemy (*H. aoronymnphium* and *L. heterotoma*, respectively; Jaenike and Brekke 2011; Xie et al. 2015). Nevertheless, the results of the present study suggest that infection by *Spiroplasma* may be weakly detrimental to larva‐to‐adult survival of *D. melanogaster* under certain conditions, but not others; for example, Xie et al. (2014) did not detect such costs, albeit under apparently equivalent experimental conditions to the present study. A cost to harboring *Spiroplasma* was also detected in *D. melanogaster* by Herren et al. (2014), where fly life span was compromised. In addition, *Spiroplasma* MSRO is a male killer, which is maintained at low frequencies in natural populations (Montenegro et al. 2005; Ventura et al. 2012). Field studies will ultimately be required to better understand the ecological and coevolutionary dynamics of *Drosophila*,* Spiroplasma,* and parasitoids.

An exciting implication of the occurrence of closely related resistant and susceptible (to *Spiroplasma*) parasitoids is that it will facilitate comparative approaches to understanding the mechanisms of protection and their evolution, which at present is fragmentary. The possible (nonmutually exclusive) mechanisms by which *Spiroplasma* may interfere with wasp growth and ultimately cause wasp death can be grouped into three categories. These categories are analogous to the following types of interspecific competition defined in classical ecology (Gerardo and Parker 2014). (1) Apparent competition: *Spiroplasma* indirectly interferes with wasp larval development by enhancing aspects of the host‐encoded immunity. (2) Exploitation competition: by competing for the same limiting resource (e.g., lipids circulating in the host's hemolymph; Paredes‐Escobar 2014), *Spiroplasma* indirectly inhibits wasp development. (3) Interference competition: by producing a substance (e.g., a ribosome inactivating protein; Hamilton et al. 2016) that is toxic to the developing wasp, *Spiroplasma* directly causes wasp death. The different wasps susceptible to *Spiroplasma* might be affected by the same or distinct specific mechanism. Similarly, wasps that are unaffected by the presence of *Spiroplasma* may achieve this by killing/incapacitating *Spiroplasma* cells (resistance per se; Ayres and Schneider 2008) or may possess tolerance to *Spiroplasma* (e.g., *Spiroplasma* densities are unaffected by the wasp, but the wasp is impervious to a *Spiroplasma* toxin).

The strategies employed by parasitoids to suppress *Drosophila* immune response are extremely diverse and rely on substances injected by the wasp during oviposition (reviewed in Heavner et al. 2013; Keebaugh and Schlenke 2013; Mortimer 2013; Colinet et al. 2014). The canonical antiwasp immune response of some, but not all, drosophilids involves melanotic encapsulation, and concomitant death, of the wasp embryo (Kacsoh et al. 2014). Depending on the wasp species (and strain), the following steps of the *Drosophila* melanotic encapsulation process are reportedly affected by one or more wasps: (1) plasmatocyte activation (*G. *sp. G1F1); (2) lamellocyte production (e.g., *L. heterotoma*,* A. citri*,* A. japonica*); (3) capsule formation (e.g., *L. boulardi*,* L. heterotoma*); and (4) capsule melanization and consolidation (e.g., *L. boulardi*,* L. heterotoma*,* A. citri*,* L. victoriae*). It is possible that *Spiroplasma* may counter one or more of the above wasp strategies, thereby partially or completely restoring host immune function. It should be noted, however, that for the two wasp species examined to date [i.e., *L. heterotoma* in *D. melanogaster* and *D. hydei*; and *L. boulardi* in *D. melanogaster*; (Xie et al. 2011, 2014; Paredes‐Escobar 2014)], *Spiroplasma*‐mediated wasp death occurs at the wasp larval stage, which is later than the stage typically killed by melanotic encapsulation. Furthermore, successful antiwasp response not involving melanotic encapsulation has been reported in drosophilids (Carton et al. 2009; Kacsoh et al. 2014). Thus, *Spiroplasma* may enhance aspects of noncanonical antiwasp mechanisms.

Comparison of the reciprocal physiological effects of *Spiroplasma* and wasps (resistant and susceptible) should lead to a comprehensive understanding of the wasp killing mechanism. The current (Hoskins et al. 2015; Paredes et al. 2015) or near future availability of sequenced genomes for all partners in these interactions (e.g., *L. heterotoma* and *G. *sp.; T. Schlenke pers. comm.), the genetic toolkit available for *D. melanogaster*, the extensive knowledge and interest on the reciprocal behavioral and physiological responses and adaptations of *Drosophila* and parasitoids (e.g., Kraaijeveld et al. 2009; Keebaugh and Schlenke 2012; Lefevre et al. 2012; Milan et al. 2012; Goecks et al. 2013; Kacsoh et al. 2013; Singh et al. 2015), and the promising recent developments in wasp RNAi (e.g., Colinet et al. 2014), will enable the integration of powerful omics and genetic manipulation approaches to identify genes and pathways relevant to the *Spiroplasma* protective mechanism and uncover their evolutionary patterns.

## Conflict of Interest

The authors declare no conflict of interests.

## Data Accessibility

Data are archived in Data Dryad doi: 10.5061/dryad.fb40c.

## Supporting information


**Table S1.** Statistical models and results for the tests of the effect of *Spiroplasma* MSRO infection on four fly or wasp survival/mortality measures.

**Data S1.** Commands and rationale for statistical analyses performed in this study.
