# Supplementary material for: Independent origins of resistance or susceptibility of parasitic wasps to a defensive symbiont
Source: Ecol Evol. 2016 Mar 16;6(9):2679–87. doi: 10.1002/ece3.2085 (PMC4798148; doi:10.1002/ece3.2085)
Supplement: Supplementary file 1 — Table S1. Statistical models and results for the tests of the effect of Spiroplasma MSRO infection on four fly or wasp survival/mortality measures. [file ECE3-6-2679-s001.docx]

| Supporting Table S1. Statistical models and results for the tests of the effect of *Spiroplasma* MSRO infection on four fly or wasp survival/mortality measures. | | | | | | | | | | | | | | | | |
| --- | --- | --- | --- | --- | --- | --- | --- | --- | --- | --- | --- | --- | --- | --- | --- | --- |
|  | **Fly over larva** | | | | **Wasp over larva** | | | | **Pupa over larva** | | | | **Failed pupa over pupa** | | | |
| **wasp treatment** | **model/ assumptions** | **d.f.^1^** | **Chi-Square or F-value^2^** | **P-value^3^** | **model/ assumptions** | **d.f.^1^** | **Chi-Square or F-value^2^** | **P-value^3^** | **model /assumptions** | **d.f.^1^** | **Chi-Square or F-value^2^** | **P-value^3^** | **model/ assumptions** | **d.f.^1^** | **Chi-Square or F-value^2^** | **P-value^3^** |
| Ajap | LOGISTIC/ FIRTH | 1 | 1.18 | 0.2783 | GLIMMIX random _residuals | 1,27 | 4.06 | 0.054 | GLIMMIX random isoline; random _residuals | 1,25 | 2.24 | 0.1466 | GLIMMIX random _residuals | 1,26 | 1.79 | 0.193 |
|  |  |  |  |  |  |  |  |  | isoline covtest | 1 | 3.43 | 0.0321* |  |  |  |  |
| G1F1 | not tested (0 survival in both treatments) |  |  |  | GLIMMIX random _residuals | 1,28 | 0.00 | 0.9918 | GLIMMIX random isoline random _residuals | 1,26 | 5.45 | 0.0275* | GLIMMIX random isoline; random _residuals | 1,26 | 0.21 | 0.6517 |
|  |  |  |  |  |  |  |  |  | isoline covtest | 1 | 0.00 | 0.4926 | isoline covtest | 1 | 0.54 | 0.2318 |
| GxHaw | LOGISTIC/ FIRTH | 1 | 9.14 | 0.0025** | LOGISTIC/ FIRTH | 1 | 46.62 | <0.0001*** | GLIMMIX random isoline random _residuals | 1,26 | 0.24 | 0.6276 | GLIMMIX random isoline random _residuals | 1,26 | 42.48 | <0.0001*** |
|  |  |  |  |  |  |  |  |  | isoline covtest | 1 | 0.19 | 0.33 | isoline covtest | 1 | 4.28 | 0.0171* |
| LgG500 | LOGISTIC/ FIRTH | 1 | 8.84 | 0.003** | GLIMMIX random isoline random _residuals | 1,26 | 0.02 | 0.8915 | GLIMMIX random _residuals | 1,28 | 0.25 | 0.6236 | GLIMMIX random isoline random _residuals | 1,26 | 0.22 | 0.6463 |
|  |  |  |  |  | isoline covtest | 1 | 0.00 | 1 |  |  |  |  | isoline covtest | 1 | 0.02 | 0.4492 |
| LvHaw | LOGISTIC/ FIRTH | 1 | 58.79 | <0.0001*** | LOGISTIC/ FIRTH | 1 | 25.78 | <0.0001*** | GLIMMIX random _residuals | 1,28 | 0.97 | 0.3322 | GLIMMIX random _residuals | 1,28 | 10.51 | 0.0031 |
| No wasp | GLIMMIX random isoline random _residuals | 1,26 | 11.80 | 0.002** | n/a |  |  |  | GLIMMIX random _residuals | 1,27 | 2.97 | 0.096 | GLIMMIX random isoline random _residuals | 1,26 | 8.35 | 0.0077** |
|  |  |  |  |  |  |  |  |  |  |  |  |  | isoline covtest | 1 | 4.41 | 0.018* |
| ^1^ degrees of freedom. For F-value: numerator, denominator | | | | |  |  |  |  |  |  |  |  |  |  |  |  |
| ^2^ Chi-Square for Logistic/Firth and Isoline covtest; otherwise F-value | | | | | |  |  |  |  |  |  |  |  |  |  |  |
| ^3^ *** significant at P<0.0001; ** significant at P<0.01; * significant at P<0.05 | | | | | |  |  |  |  |  |  |  |  |  |  |  |
