# Supplementary material for: Independent origins of resistance or susceptibility of parasitic wasps to a defensive symbiont
Source: Ecol Evol. 2016 Mar 16;6(9):2679–87. doi: 10.1002/ece3.2085 (PMC4798148; doi:10.1002/ece3.2085)
Supplement: Supplementary file 2 — Data S1. Commands and rationale for statistical analyses performed in this study. [file ECE3-6-2679-s002.docx]

Supporting Data for “**Independent origins of resistance or susceptibility of parasitic wasps to a defensive symbiont”**

Mariana Mateos^1,*^, Lauryn Winter^1^, Caitlyn Winter^1^, Victor M. Higareda-Alvear^2^, Esperanza Martinez-Romero^2^, Jialei Xie^1^

Statistical Analyses

SAS commands used for implementation of the logisitic regression with penalized likelihood (Firth) analyses (King and Zeng, 2001). These were implemented for datasets in which the number of observations in one category was zero or close to zero (e.g. zero larva-to-adult fly survival).

------------------------------------------------------------------------------------------------------------

ods graphics on;

PROC Logistic DATA=WORK.FILTER_FOR_WASP_PROTECTION_ROUND; *plots=all;

BY wasp_name ;

CLASS SpiroYN Isoline ;

*MODEL total_fly/real_larvae = SpiroYN / firth;

*MODEL total_wasp/real_larvae = SpiroYN / firth;

*MODEL Pupae/real_larvae = SpiroYN / firth;

MODEL failed_pupae/Pupae = SpiroYN / firth;

run;

ods graphics off;

------------------------------------------------------------------------------------------------------------

The commands for the generalized linear mixed model with binomial distribution, including a Covtest (ratio of pseudo-likelihoods) for the random variable isoline were as follows.

------------------------------------------------------------------------------------------------------------

ods graphics on;

*Random _residual_ and isoline;

proc glimmix data=FILTER_FOR_WASP_PROTECTION_ROUND plot=studentpanel ;

BY wasp_name ;

CLASS SpiroYN Isoline;

*MODEL total_fly/real_larvae = SpiroYN / dist=binomial solution cl;

*MODEL total_wasp/real_larvae = SpiroYN / dist=binomial solution cl;

*MODEL Pupae/real_larvae = SpiroYN / dist=binomial solution cl;

MODEL failed_pupae/Pupae = SpiroYN / dist=binomial solution cl;

Random Isoline /s;

Random _Residual_;

covtest 'isoline ' 0 . ./cl;

output out=gmxout pred=pred pred(ilink)=predmu pearson=pearson;

run;

proc means data=gmxout n mean var;

var pearson;

run;

ods graphics off;

------------------------------------------------------------------------------------------------------------

If the above analyses did not converge or if the G-matrix was not positive definite, then we implemented an analysis without regard to the isoline variable with the following commands.

------------------------------------------------------------------------------------------------------------

ods graphics on;

proc glimmix data=FILTER_FOR_WASP_PROTECTION_ROUND plot=studentpanel ;

BY wasp_name ;

CLASS SpiroYN Isoline;

*MODEL total_fly/real_larvae = SpiroYN / dist=binomial solution cl;

*MODEL total_wasp/real_larvae = SpiroYN / dist=binomial solution cl;

*MODEL Pupae/real_larvae = SpiroYN / dist=binomial solution cl;

MODEL failed_pupae/Pupae = SpiroYN / dist=binomial solution cl;

Random _Residual_;

output out=gmxout pred=pred pred(ilink)=predmu pearson=pearson;

run;

proc means data=gmxout n mean var;

var pearson;

run;

ods graphics off;

------------------------------------------------------------------------------------------------------------

The following commands were used to sort the dataset by wasp treatment prior to the statistical analyses.

------------------------------------------------------------------------------------------------------------

PROC SQL;

CREATE TABLE WORK.FILTER_FOR_WASP_PROTECTION_ROUND AS

SELECT t1.Isoline,

t1.Wasp,

t1.wasp_name,

t1.Date,

t1.Larvae,

t1.Oviposition,

t1.Pupae,

t1.total_fly,

t1.'fly/larvae'n,

t1.'fly/pupae'n,

t1.total_wasp,

t1.'wasp/larvae'n,

t1.'wasp/pupae'n,

t1.'total adults'n,

t1.'adult/pupae'n,

t1.'failed pupae prop'n,

t1.'pupae/larvae'n,

t1.real_larvae,

t1.failed_pupae,

t1.SpiroYN

FROM WORK.WASP_PROTECTION_ROUND_2_SIMPLIFI t1

ORDER BY t1.wasp_name;

QUIT;

------------------------------------------------------------------------------------------------------------

**Literature Cited**

King, G., and L. Zeng. 2001. Logistic regression in rare events data. Political Analysis 9:137-163.
